# Prediction and Experimental Study of Low-Frequency Acoustic and Vibration Responses for an Aircraft Instrument Compartment Based on the Virtual Material Method

**DOI:** 10.3390/ma18050932

**Published:** 2025-02-20

**Authors:** Shaowei Song, Jun Wang, Chang Liu, Rongze Huang

**Affiliations:** 1 School of Aerospace Engineering, Xi’an Jiaotong University, Xi’an 710049, China; songshw82@163.com; 2 National Key Laboratory of Aerospace Liquid Propulsion, Xi’an Aerospace Propulsion Institute, Xi’an 710100, China; junw83@163.com (J.W.); 13720753215@163.com (C.L.); 3 State Key Laboratory for Strength and Vibration of Mechanical Structures, School of Mechanical Engineering, Xi’an Jiaotong University, Xi’an 710049, China

**Keywords:** aircraft instrument compartment, virtual material method, modal testing, vibration and noise

## Abstract

Bolted connections are extensively utilized in aircraft structures, and accurately simulating these connections is a critical factor affecting the precision of vibration and noise response predictions for aircraft. This study focuses on an instrument compartment of a specific aircraft model, employing the virtual material method to simulate the bolted joints within the structure. Parameters for the virtual material layer were obtained through theoretical calculations combined with parameter identification methods, achieving precise modeling of the instrument compartment. By comparing the calculated modes with the experimental modes of the instrument compartment, it was found that the first four modal shapes from both calculation and experiment were completely consistent, with the error in natural frequencies within three percent. Subsequently, acoustic and vibration computations were performed using both the virtual material model and the tied constraint model, with comparisons made against experimental results. The findings indicate that the root mean square (RMS) acceleration response of the virtual material model was 11.23 g, closely matching the experimental value of 10.35 g. Additionally, the total sound pressure level inside the acoustic cavity was 136.98 dB, closely aligning with the experimental value of 135.76 dB. These results demonstrate that the virtual material method offers higher accuracy in structural acoustic and vibration calculations.

## 1. Introduction

Bolted connections are prevalent in various aircraft structures, but the complex mechanisms at their interfaces can lead to nonlinear dynamic phenomena such as energy dissipation, stiffness softening, and increased local damping at the bolted interfaces [1,2,3], which can influence the entire structure’s modal and transfer function characteristics [4]. Precise models are required to describe the dynamic characteristics of significant and complex equipment like aircraft devices.

During high-speed flight, the interaction between high-speed fluid and the wall surface forms a turbulent boundary layer on the aircraft’s surface, resulting in powerful aerodynamic fluctuation pressures and inducing an aerodynamic noise field with frequencies reaching up to 8000 Hz. This high-frequency noise strongly couples with the aircraft structure, producing vibrations with root-mean-square (RMS) acceleration responses as high as 50 g [5], subjecting the aircraft structure and internal instruments to complex and harsh environments. To understand the response levels and reveal the response patterns to improve the aircraft’s resistance to acoustic and vibrational effects, simulation predictions and ground-based acoustic and vibration tests must be conducted [6,7,8]. The dynamic response calculation of structures with nonlinear characteristics is a complex problem. Karpenko et al. [9] obtained reliable nonlinear material properties by combining experimental research with numerical simulations. Due to the wide frequency spectrum of the noise environment, it is challenging to predict the full-band acoustic and vibration responses using a single method [10]. Typically, the frequency band is divided into low, medium, and high segments, with the finite element method used for the low-frequency band, statistical energy analysis for the high-frequency band, and hybrid methods for the mid-frequency band. Given the numerous components and complex interconnections of aircraft structures, accurately simulating joint connections is crucial for predicting acoustic and vibration behavior.

Currently, scholars have achieved fruitful results in modeling structural joints, with common bolted interface characterization methods including the Iwan equivalent model [11,12,13], thin-layer element method [14,15,16], and virtual material method [17,18,19], among others, the latter being widely applied due to its high modeling accuracy and low computational cost. Although equivalent models of bolted joints can accurately represent the nonlinear dynamic characteristics of simple connection structures, research applying this theory to large, complex assembled structures like aircraft for acoustic and vibration issues is relatively scarce. As the modeling of bolted joints significantly impacts structural dynamics, further investigation into its influence on acoustic and vibration prediction problems is necessary. This study uses an aircraft instrument compartment model as the object of research, establishing models using both the virtual material method and the tied constraint method for predicting low-frequency band acoustic and vibration responses under specified noise excitation conditions, followed by experimental validation. Since the finite element method is suitable for low-frequency band acoustic and vibration response predictions, considering the model’s characteristics, the calculation frequency band is selected as 50–400 Hz, investigating the impact of micro-contact characteristics of bolted joints on the low-frequency band acoustic and vibration prediction results, aiming to enhance the precision of low-frequency band acoustic and vibration predictions.

## 2. Parametric Modeling of Structural Bolted Joint Virtual Material Layer Dynamics

### 2.1. Basic Principles of Assumptions in the Virtual Material Method

The virtual material method involves adding a layer of virtual material between two component bolted joints, simulating the dynamic characteristics of the bolted joint by altering parameters such as the density, elastic modulus, and Poisson’s ratio of the virtual material [20]. The isotropic virtual material theory posits that the interface has varying degrees of roughness, which can be considered as a whole, formed by microasperities distributed according to different features, with the heights of these microasperities following a normal distribution, and being isotropic, with the essence of rough interface contact being the deformation of microasperity contacts.

The parameters of the virtual material layer include elastic modulus E, Poisson’s ratio µ, thickness h, and density ρ. These parameters are related to the material properties of the two components and the contact surface data (such as surface roughness, bolt preload, etc.).

### 2.2. Determination of Virtual Material Layer Parameters for Structural Bolted Joints

#### 2.2.1. Determination of Virtual Material Layer Thickness and Density

The thickness *h* of the virtual material layer is the sum of the thicknesses of two layers of microasperities on the rough surfaces, calculated using the formula:

(1) $$h=h_{1}+h_{2}$$

where $h_{1}$ and $h_{2}$ are the thicknesses of the microasperity layers on the contact surfaces of the two parts, which are typically 1 mm.

According to the definition of material density, the density *ρ* of the virtual material can be obtained by the following equation:

(2) $$\rho =\frac{m_{1}+m_{2}}{V_{1}+V_{2}}=\frac{\rho_{1}A_{a}V_{1}+\rho_{2}A_{a}V_{2}}{A_{a}\left(h_{1}+h_{2}\right)}=\frac{\rho_{1}h_{1}+\rho_{2}h_{2}}{h_{1}+h_{2}}$$

where $A_{a}$ is the nominal contact area of the two connectors.

Thickness and density can be relatively simply derived from formulas. However, the calculation of the elastic modulus and Poisson’s ratio is more complex and theoretical calculations often contain some error. In engineering applications, parameter identification methods are frequently used to obtain these values [21].

#### 2.2.2. Parameter Identification for Elastic Modulus and Poisson’s Ratio of the Virtual Material Layer

**Figure 1:**
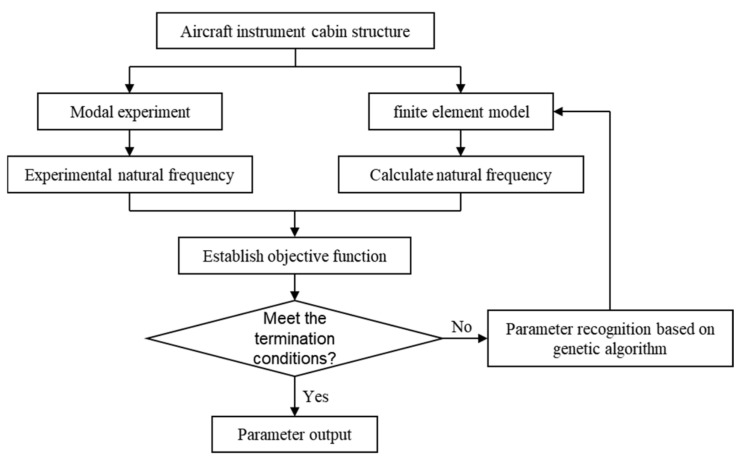
Flowchart of virtual material layer parameter identification.

Parameter identification involves iteratively adjusting model parameters so that the computational results approach experimental results, ultimately obtaining an optimal set of parameters within an acceptable error range. The specific identification process is illustrated in Figure 1. The basic steps for parameter identification in this paper are as follows:

1. Establish a finite element model of the aircraft instrument compartment structure in Ansys, incorporating a virtual material layer at the bolted joints, and perform simulation calculations to obtain initial computed frequencies and mode shapes.
2. Conduct modal experiments on the aircraft instrument compartment to acquire actual natural frequencies and mode shapes of the structure.
3. Formulate an objective function based on the structural computed frequencies and the experimental frequencies obtained from modal testing, with the elastic parameters of the virtual material layer serving as design variables.
4. Set constraints and apply genetic algorithms to identify the elastic parameters of the virtual material layer.
5. Once the objective function meets the termination criteria, the identified parameters for the virtual material layer model are obtained.

The objective function of parameter recognition is defined as the finite element calculation and the natural frequency difference obtained by the modal experiment is minimized, as shown in the following equation:

(3) $$F_{ul}=min\overset{3}{\underset{j=1}{\sum }}{\left(\frac{f_{j}^{a}}{f_{j}^{e}}-1\right)}^{2}$$

In the formula: $f_{j}^{a}$ represents the calculated natural frequency; $f_{j}^{e}$ represents the experimental natural frequency; The objective function is defined to minimize the differences between the first three experimental frequencies and the calculated frequencies of the aircraft compartment. The accuracy of the parameter identification for the equivalent model of the virtual connection layer is validated using the fourth natural frequency, which was not involved in the identification process.

## 3. Modal Acquisition of Instrument Compartment and Identification of Elastic Parameters of Virtual Material Layer

The instrument compartment model of the aircraft discussed in this paper features a conical shell structure with a thickness of 5 mm. The lower part is sealed with a circular plate, and the upper part is closed by a second-order curved surface plate. The material used is aluminum alloy, and it includes two bolted interfaces, each connected by six uniformly distributed M16 bolts. The surface roughness of any two component surfaces is considered in the design. According to the parameter identification method presented in this paper, it is necessary to obtain both the experimental modes and calculated modes of the aircraft instrument compartment. By iteratively adjusting the elastic parameters of the virtual material layer, the optimal set of parameters is ultimately obtained.

### 3.1. Modal Test of Aircraft Instrument Compartment

**Figure 2:**
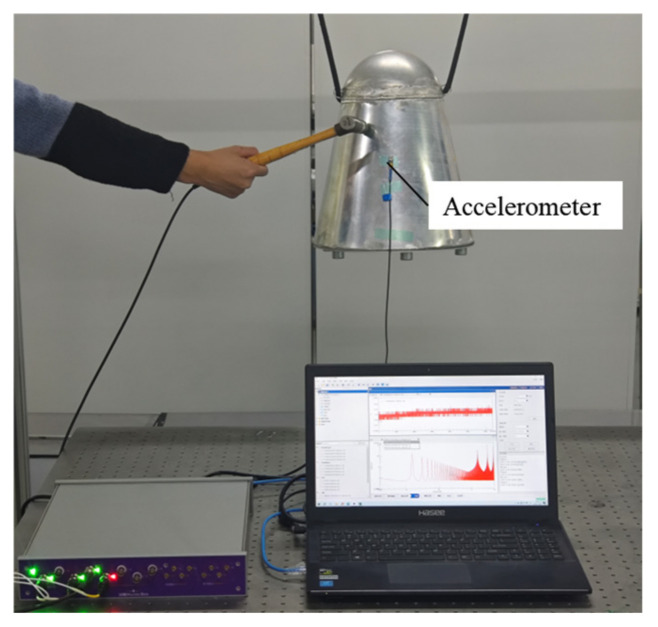
Modal experiment of aircraft instrument cabin.

To conduct free modal testing on the aircraft instrument compartment model, it was suspended as shown in Figure 2. The modal experiment employed the impact hammer method for pulse excitation to obtain the structure’s free modes. Considering the structural characteristics, 193 equidistant measurement points were arranged on the second-order curved plate and the conical shell body. One accelerometer is arranged on the structure surface. Since the mass of the accelerometers is much smaller compared to the model mass, their influence on the test can be neglected. Each measurement point was measured four times to ensure data reliability. The Hunter Box (Hanhang (Beijing) Technology Co., Limited, Beijing, China) data acquisition system recorded the experimental data and performed modal analysis. The modal shapes and frequency data obtained will be presented in the following sections for comparative purposes.

### 3.2. Modal Calculation of the Instrument Compartment of the Aircraft

**Figure 3:**
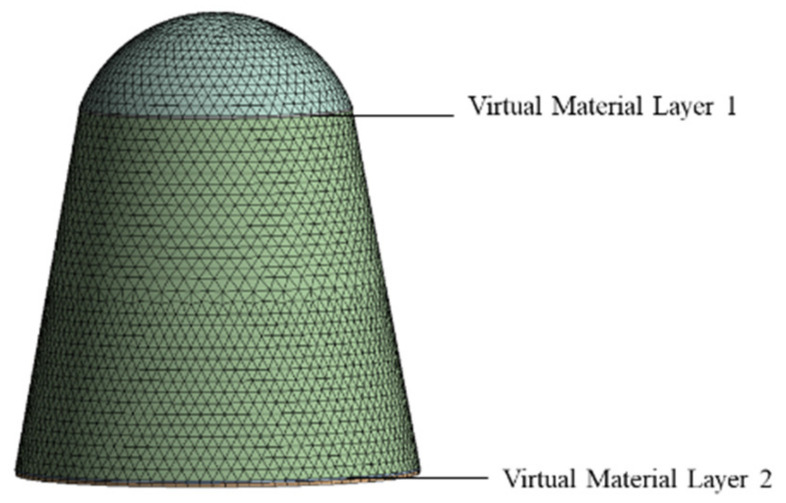
Finite element model of the virtual materials method instrument compartment.

When establishing the finite element model of the instrument compartment, we removed the bolts top hooks, and other parts that have little influence on the structural dynamics, and retained the bolt holes. The finite element model of the instrument compartment obtained based on the virtual material method is shown in Figure 3, with a minimum grid size of 5 mm, including 114,049 elements and 288,505 nodes. The model was analyzed in free mode.

### 3.3. Identification of Elastic Parameters of Structural Virtual Material Layer Based on Genetic Algorithm

**Figure 4:**
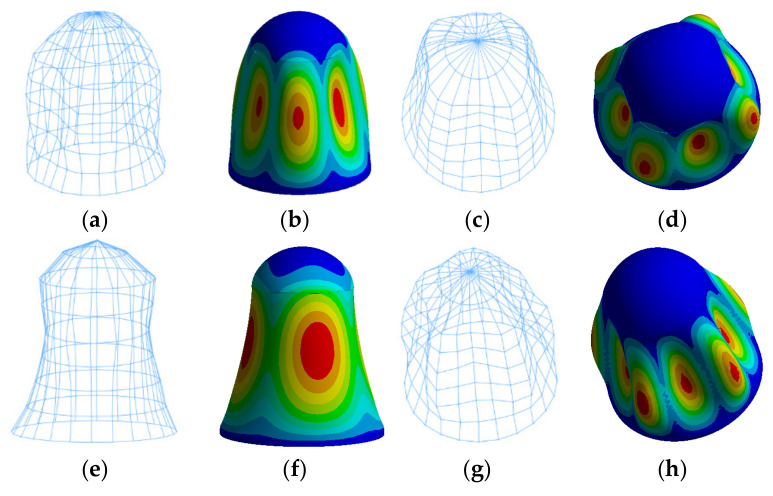
(a) 1st order test mode shape; (b) 1st order simulated mode shape; (c) 2nd order test mode shape; (d) 2nd order simulated mode shape; (e) 3rd order test mode shape; (f) 3rd order simulated mode shape; (g) 4th order test mode shape; (h) 4th order test mode shape.

The comparison of the first four modal shapes of the aircraft instrument compartment obtained from experiments and calculations is shown in Figure 4. The calculated modal shapes are fundamentally consistent with the experimental modal shapes. On the premise of consistent modal shapes, according to Equation (2), the objective function for the genetic algorithm is defined as the minimization of the differences between the experimental and simulated values of the structure’s first three natural frequencies. With each iteration of the genetic algorithm, the updated elastic modulus and Poisson’s ratio after iteration are applied to the connection layer model. The process continues until the termination criteria are met.

The initial values and ranges of variation for the material parameters are provided in Table 1.

After calculation, the objective function converges after 22 iterations, and 20 design points are calculated for each iteration. The elastic parameters of the virtual material layer of the structure after parameter identification are shown in Table 2. The calculated modal frequencies obtained by the virtual material model and the binding constraint model are compared with the experimental modal frequencies, as shown in Table 3. The frequency error of the first three modes of the model obtained by using the virtual material method is less than 3%, and the calculation accuracy is high, and the error of the fourth natural frequency that does not participate in the recognition is also less than 3%, which verifies the accuracy of the parameter identification method. Compared with the traditional binding constraint method, the modal frequency error obtained by the virtual material method is lower, which indicates that the virtual material method has higher accuracy than the binding constraint method.

## 4. Calculation and Test Comparison and Analysis of the Acoustic and Vibrating Response of the Instrument Compartment

**Figure 5:**
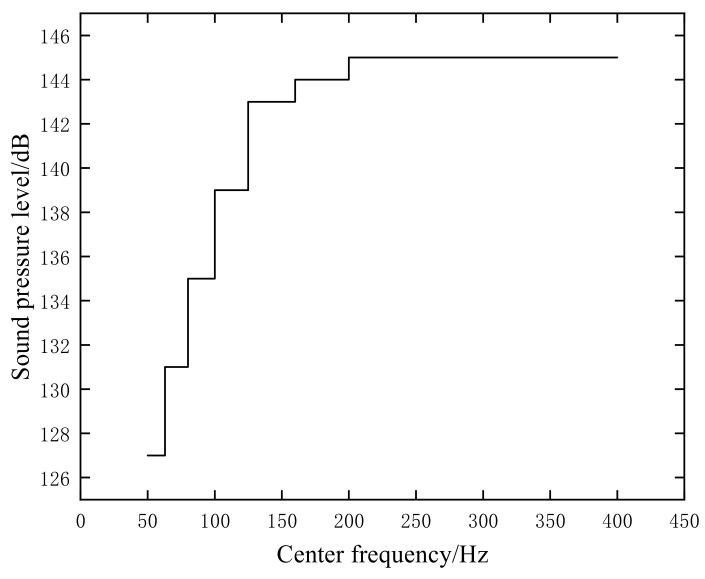
Experimental spectrum of the external acoustic field.

To compare the differences in computational results between the virtual material method and the tied constraint method, models of the instrument compartment are created using both approaches. The vibration and noise responses are then analyzed based on these models. The mesh models are imported into acoustic and vibration analysis software, and the frequency band from 50 to 400 Hz, as shown in Figure 5 according to environmental test standard spectra, is selected as the noise excitation spectrum for both computation and experimentation. The calculated values of the structural acoustic and vibration responses are obtained through this process.

**Figure 6:**
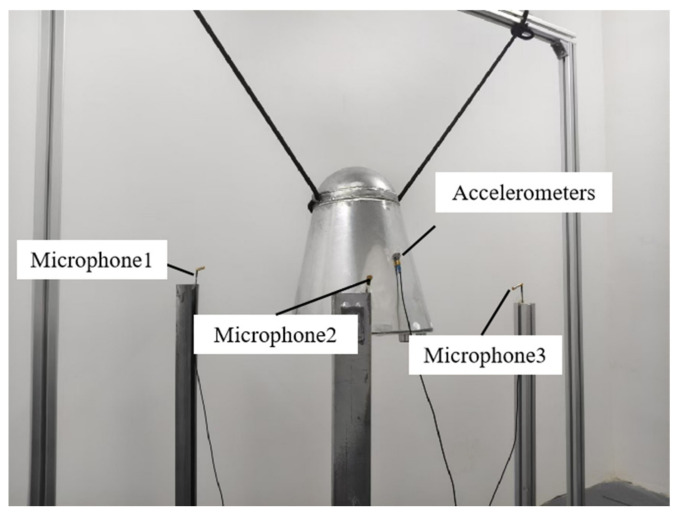
Model noise excitation test site.

The experiment in this article is based on the acoustic control module of the HANHANG testing system and conducted in a simple reverberation room at Xi’an Jiaotong University. The volume of the reverberation room is 70 m^3^, and the walls are made of high-density concrete, which can effectively reflect sound waves. The experiment measured the structural vibration response and the internal noise response under a specified noise spectrum excitation, with a noise exposure duration of 30 s. The model was suspended within the reverberation chamber using rubber cords. Three microphones were arranged around the model to employ a three-point averaging method for closed-loop control of the acoustic field. The test site is illustrated in Figure 6.

### 4.1. Comparison of the Vibration Response Calculation and Test Results of the Instrument Compartment

**Figure 7:**
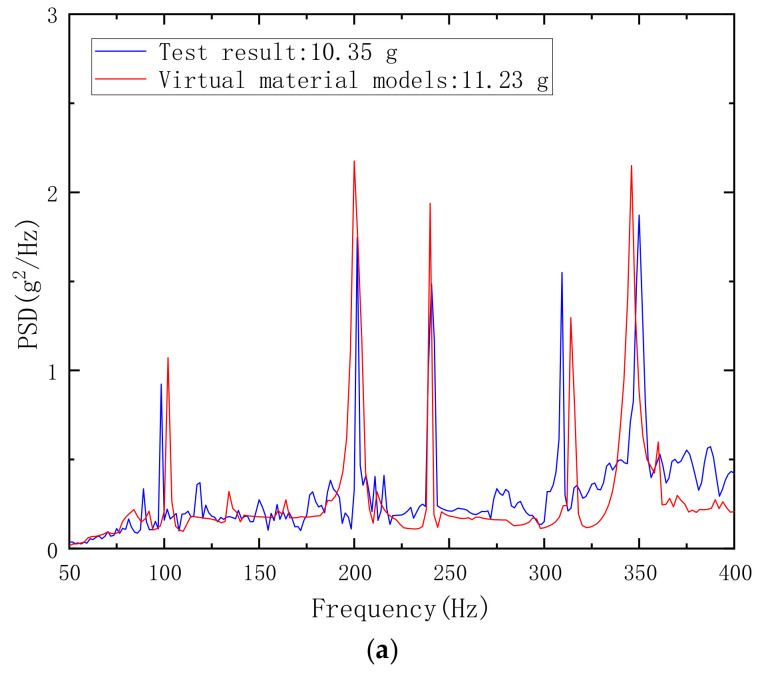

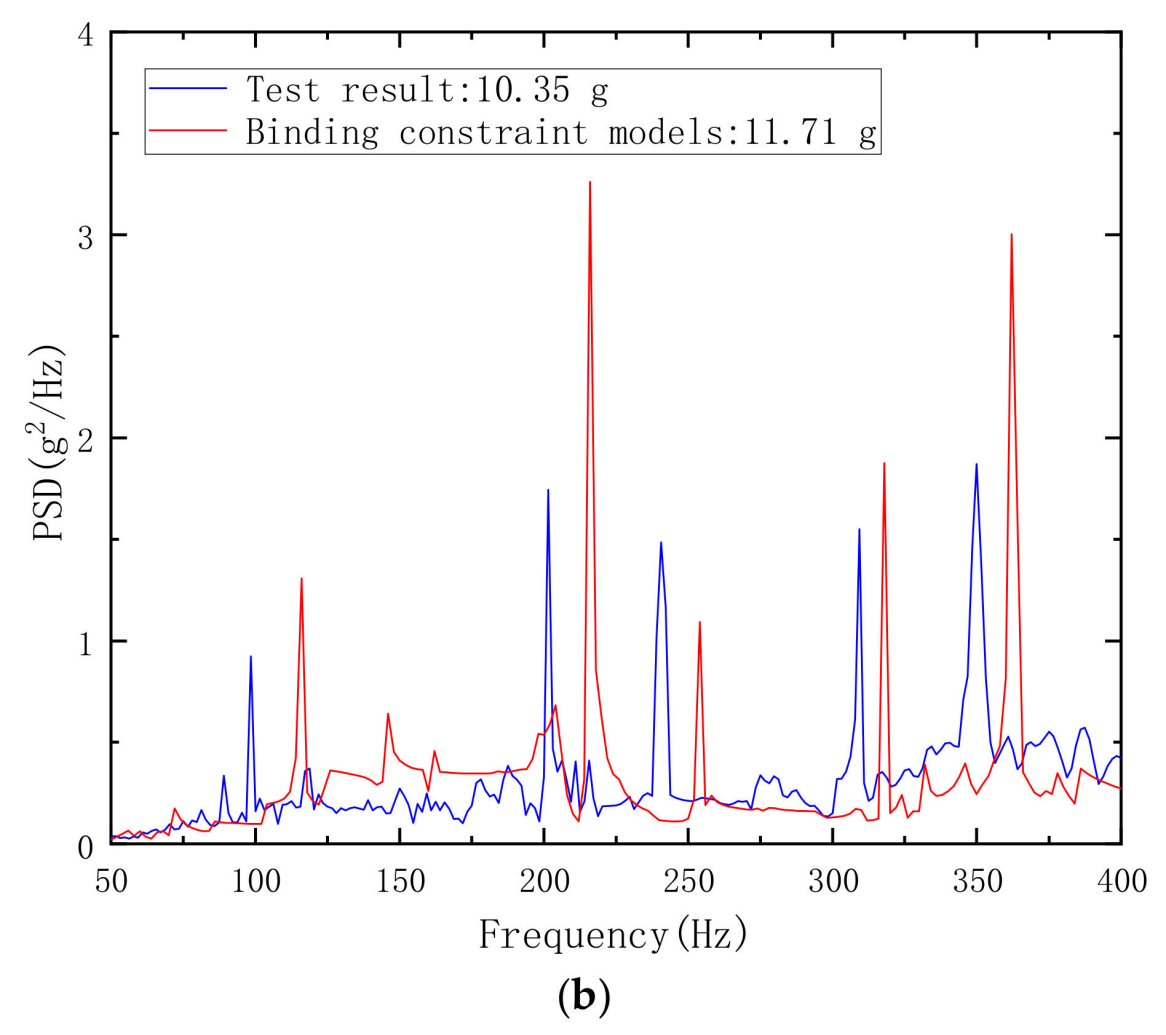
(**a**) Comparison of Calculated and Experimental Values of Acceleration PSD (Virtual Material Method); (**b**) Comparison of Calculated and Experimental Values of Acceleration PSD (Binding Constraint Method).

Figure 7 shows the comparison of the calculated acceleration power spectral density curves of the two models with the experimental measured acceleration power spectral density curves, and gives the root mean square value of acceleration. As seen in Figure 7, the acceleration power spectral density curve calculated based on the virtual material model is closer to the experimental values, and the frequency position of the vibration peak is very close. Table 4 shows the frequency of the peak in the experimental and simulated response curves, as well as the frequency offset and error percentage of the simulated values relative to the experimental values. The frequency offset of the virtual material model is much lower than that of the binding constraint model, not exceeding 7 Hz, while the frequency offset of the third peak point of the binding constraint model exceeds 20 Hz. This is caused by the natural frequency of the computational model. The binding constraint law rigidly connects the joint surface, increasing the structural stiffness and natural frequency accordingly. In addition, the vibration peak of the virtual material model is also closer to the actual value. The root mean square value of acceleration obtained from the experiment is 10.35 g, and the root mean square values of acceleration calculated by the virtual material model and the binding constraint model are 11.23 g and 11.71 g, respectively. Obviously, the accuracy of the virtual material model is higher.

### 4.2. Calculation of Noise Response of Acoustic Cavity Inside the Instrument Compartment and Comparison of Test Results

Table 5 shows the comparison of the calculated and experimental values of the two modeling methods within the 1/3 octave range of the sound pressure level of the internal acoustic cavity. At each center frequency point, the sound pressure level obtained based on the virtual material method is closer to the test value, and the total sound pressure level error is not more than 2 dB. The result obtained by the binding constraint method is larger, and the total sound pressure level error is 2.97 dB, because the binding constraint method makes the structural connection surface rigidly connected, which will amplify the noise response to a certain extent.

## 5. Conclusions

This paper focuses on the instrument compartment of a specific aircraft model, employing the virtual material method for precise modeling. The virtual material parameters for the bolted joints were obtained through theoretical calculations and parameter identification, further analyzing the impact of joint contact characteristics on the prediction of structural vibration and noise. The main conclusions are as follows:

1. Accurate Simulation with Virtual Material Method: By adding a layer of virtual material at the bolted interfaces, the virtual material method can accurately simulate the contact characteristics of the joints. While theoretically calculating the elastic parameters (elastic modulus, Poisson’s ratio) of the virtual material is complex, using parameter identification provides a simpler approach to obtaining these parameters with high precision.
2. High Precision in Modal Analysis: For structures modeled using the virtual material method, the calculated modal shapes are consistent with experimental modal shapes, and the error between computed frequencies and experimental frequencies is within 3%. Compared to the tied constraint method, this approach offers higher accuracy, indicating that the virtual material method better approximates real-world conditions when simulating bolted connections.
3. Superior Vibration and Acoustic Prediction: The structure modeled using virtual material method has a smaller frequency offset of peak vibration response, not exceeding 7 Hz, while the peak frequency offset of the bound constraint model exceeds 20 Hz; The root mean square value of the vibration response acceleration of the virtual material model is also closer to the experimental results. In terms of noise response, the sound pressure level error at each center frequency point is smaller, and the total sound pressure level error does not exceed 2 dB. The above results indicate that the virtual material method is more accurate in describing the dynamic characteristics of structures.

## Figures and Tables

**Table 1:** Initial values and variation range of virtual material layer parameters.

Material Parameters	Initial Value	Lower Limit	Upper Limit
Elastic modulus/GPa	70	0.1	80
Poisson’s ratio	0.3	0.15	0.45

**Table 2:** Model virtual material layer elastic parameters.

	Elastic Modulus/GPa	Poisson’s Ratio
Connection Layer 1	4.836	0.287
Connection Layer 2	3.556	0.265

**Table 3:** Instrument compartment calculation and test modal frequency comparison.

Order	Test Modal Frequency/Hz	Virtual Materials Method		Binding Constraint Method	
		Calculate Modal Frequency/Hz	Error %	Calculate Modal Frequency/Hz	Error %
1	98.44	101.24	2.84	105.61	7.28
2	201.56	204.66	1.54	217.81	8.06
3	240.63	246.79	2.56	258.51	7.43
4	309.38	315.85	2.09	330.23	6.74

**Table 4:** Comparison of Peak Frequency Calculation and Experiment between Two Models.

Test Peak Frequency/Hz	Virtual Materials Method			Binding Constraint Method		
	Calculate Peak Frequency/Hz	Frequency Offset/Hz	Error %	Calculate Peak Frequency/Hz	Frequency Offset/Hz	Error %
97.52	102.86	5.34	5.48	105.87	8.09	8.30
200.32	198.87	−1.45	−0.72	216.96	17.49	8.73
238.45	237.01	−1.44	−0.60	256.43	21.5	9.02
307.89	314.43	6.54	2.12	328.55	15.8	5.13
349.76	346.67	−3.09	−0.88	361.87	14.87	4.25

**Table 5:** Comparison of sound pressure level calculation and test in sound cavity.

Center Frequency/Hz	Sound Pressure Level in the Internal Acoustic Cavity/dB		
	Test Values	Virtual Materials Method	Binding Constraint Method
50	108.4	110.4	113.2
63	112.2	113.7	114.9
80	116.7	118.4	119.7
100	119.3	121.3	122.5
125	122.5	124.8	126.9
160	124.2	125.4	129.1
200	127.5	128.2	130.7
250	128.7	129.9	131.1
315	129.9	130.7	131.9
400	129.5	131.1	132.6
Total sound pressure level	135.76	136.98	138.73

## Data Availability

The data provided in this study can be provided at the request of the corresponding author. The research data presented in the paper consists of simulation and experimental results. We have provided the necessary simulation settings and experimental setups within the paper to enable scholars to replicate our results. Therefore, it is not necessary for us to specifically upload the data. Additionally, we have not uploaded the data from the paper to any public datasets. However, if there is a need, we are willing to provide the data upon request.
